# Diffuse Idiopathic Skeletal Hyperostosis of Cervical Spine with Dysphagia—Molecular and Clinical Aspects

**DOI:** 10.3390/ijms22084255

**Published:** 2021-04-20

**Authors:** Mikołaj Dąbrowski, Łukasz Kubaszewski

**Affiliations:** Adult Spine Orthopaedics Department, Poznan University of Medical Sciences, 61-545 Poznan, Poland; eklinika@o2.pl

**Keywords:** cervical spine, DISH, diffuse idiopathic skeletal hyperostosis, Forestier disease, dysphagia, molecular and genetical factors, DISHphagia

## Abstract

Diffuse idiopathic skeletal hyperostosis (DISH) is a condition characterized by the calcification and ossification of the ligaments of the cervical spine; in some cases, it may result in dysphagia. The condition is more common in men over 50 years of age with metabolic disorders, and it is often asymptomatic and not a major issue for patients. The etiology of DISH is poorly understood, and known genetic factors indicate multiple signal pathways and multigene inheritance. In this review, we discuss the epidemiological, clinical, and etiological aspects of DISH with a special focus on dysphagia.

## 1. Introduction

Diffuse idiopathic skeletal hyperostosis (DISH/Forestier’s disease) is a condition characterized by the calcification and ossification of the ligaments of the cervical spine, and the condition may be exclusive to this area of the spine [1].

DISH occurs with a frequency of 2–4% in patients over 40 years of age, up to 11% with middle-aged patients, and this increases to 28% in those over 80 years of age [2]. DISH is more common in men [2,3].

DISH is often confused with related major diseases such as ankylosing spondylitis, spondylosis deformans, and degenerative spinal disease [3].

## 2. Etiopathogenesis—Molecular Aspects

Little is known about the pathogenesis of DISH. The condition is associated with old age, males, obesity, hypertension, atherosclerosis, and diabetes mellitus [4]. On the molecular level, the condition is associated with genetic factors, changes in signaling pathways, and metabolic and vascular [5] inflammatory [6] factors.

The pathogenesis of DISH is likely polygenic and dependent on the interaction of multiple gene variants, epigenetics, and environmental factors. DISH is likely influenced by many polymorphisms influencing inheritance, pathology, and expression in various genes [7].

### 2.1. Genetic Factors

Many proteins and signaling pathways are responsible for bone formation and healing, including the Wnt/β-catenin signaling pathway. Wnt ligands bind to the receptor complex (frizzled and low-density lipoprotein receptor-related protein (LRP5 or LPR6)), causing the release of β-catenin by phosphorylation, which releases it from the multiprotein complex. The accumulation of β-catenin in the cytoplasm and ultimately its translocation to the nucleus result in the activation of target genes [8]. TGF-β is another signaling pathway involved in the differentiation of bone-forming osteoblasts, skeletal genesis, and bone homeostasis. In humans, heterozygous pathogenic changes in fibrillin 1 (FBN1), transforming growth factor beta receptor 1/2 (TGFBR1/2), or in TGF-β inhibit this signaling pathway, phenotypically causing skeletal-deformity diseases (Marfan, Loeys Dietz, and Camurati–Engelman syndrome) [9]. Many cytokines, growth factors, hormones, and vitamins are involved in the bone-repair and -remodeling phases. Growth factors belonging to the TGF-β superfamily, bone morphogenetic proteins (BMP), or TGF-β1 act locally on bone formation by stimulating the proliferation and osteogenic differentiation of mesenchymal stem cells (MSC) [9].

Gorman et al. described a family with a genetic predisposition to DISH of the cervical spine [10]. Among animals, some dog breeds (for example, boxers) were found to have a higher incidence of DISH (40%) than that of other breeds (4%) [11]. Previous research found that mouse modeling lacked the equivalent volume of nucleoside transporter 1 (ENT1) signaling DISH [12].

**Figure 1:**
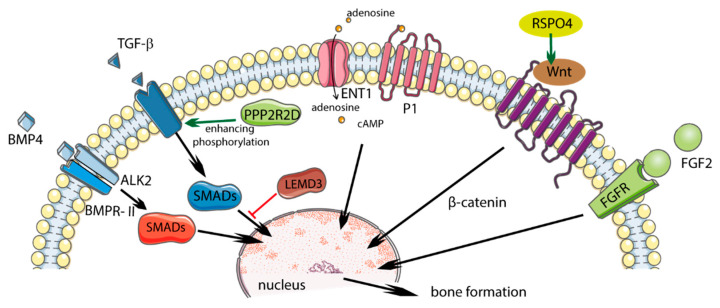
Potential role of polymorphism in diffuse idiopathic skeletal hyperostosis (DISH) and influence on regulatory pathways. Solid green arrows indicate activation; solid red bars indicate inhibition. Figure created using Servier Medical Art: https://smart.servier.com (accessed on 4 February 2021) [17].

Single nucleotide polymorphisms in the *COL6A1* and *FGF2* genes were associated with the presence of DISH [12]. Other potential genes that were studied in patients with DISH and chondrocalcinosis (DISH/CC) include R-spondin 4 (*RSPO4*), LEM domain-containing 3 (*LEMD3*), and protein phosphatase 2 regulatory subunit beta delta 2 (*PPPR2RD*) (Table 1, Figure 1) [13,14].

Future molecular studies should include other variants of the *BMP4* gene (rs17563) and the overexpression of ABCC6 [7].

#### 2.1.1. COL6A1

The α-chain of Type VI collagen is a protein of the extracellular matrix encoded by the *COL6A1* gene, which forms the basis of osteoblastic cells or chondrocytes, which participate in membrane or endochondral ossification. Certain *COL6A1* variants were shown to increase the posterior longitudinal ligament’s (OPLL) susceptibility to ossification. Single-nucleotide polymorphisms (SNPs) in *COL6A1* were only found in the Japanese DISH patient population [16]. Patients with both OPLL and DISH showed more potent *COL6A1* variants than patients with isolated OPLL did [12].

#### 2.1.2. FGF2

Fibroblast growth factor 2 (FGF2) is part of a large family of growth factors that play a role in the angiogenesis and mitogenesis of many cell types. FGF2 transmits signals through two (FGFR2 and FGFR3) of the four known FGF receptors (FGFR), which contain an extracellular immunoglobulin-like domain and a cytoplasmic tyrosine kinase domain. The FGF family of proteins are involved in stem-cell growth, tissue regeneration, and carcinogenesis [18]. The signaling pathways of FGF/FGFR play an important role in bone development. In addition, FGF signaling is involved in the maintenance of adult bone homeostasis and fracture healing. The synergism of FGF2 action with BMP2 with canonical Wnt signaling was demonstrated [19].

FGF/FGFR plays a major role in bone development, including in the pathogenesis of human ligament ossification. Previous research showed that the variant of *FGFR1* is significantly associated with OPLL, and another variant in *FGF2* is associated with conditions coexisting with DISH [16].

#### 2.1.3. RSPO4

Proteins from the R-spondin (RSPO) family consist of four members (RSpo1–4) whose structures are 60% similar. The RSPO4 protein is involved in the activation of the Wnt/β-catenin signaling pathways. The activation of the Wnt signaling pathway causes the production of proteins responsible for bone formation, while disabling this pathway reduces the production of bone tissue. In patients with DISH/CC, two variants of the *RSPO4* gene (rs146447064 and rs14915407) were found, and they occurred more often than in the control group. Reducing the expression of the *RSPO4* gene decreases Wnt activation and may “protect” against the supergrowth of new bone [14].

#### 2.1.4. LEMD3

The *LEMD3* gene encodes 60 kD inner nuclear membrane protein Man1 (LEMD3) and is a nuclear membrane protein that can inhibit the TGFβ signaling pathway by binding to SMAD proteins, which are downstream pathway mediators. The heterozygous loss of function mutations in *LEMD3* enhances TGF-β signaling and leads to bone developmental disorders such as osteopoikilosis and melorheostosis. LEMD3 normally antagonizes the activation of the bone lining to bone-forming osteoblasts, generating a modeling-based formation, possibly by reducing BMP and/or TGF-β signaling. Pathogenic *LEMD3* variants can disrupt the antagonistic effect of LEMD3 on TGF-β and BMP signaling, resulting in the increased proliferation and early differentiation of bone-lining cells [20].

A previously discovered rare *LEMD3* variant (rs201930700) of the DISH/CC phenotype may promote enhanced TGF-β signaling, leading to increased bone formation [14].

#### 2.1.5. PPP2R2D

Protein phosphatases influence the signaling of the TGF-β superfamily, which regulates numerous cellular responses. They catalyze the removal of phosphate groups from serine and/or threonine residues by the hydrolysis of phosphoric acid monoesters. PPP2R2D protein inhibits Type I ALK4 and ALK5 receptors in human cell lines, causing the blockage of TGF-β pathway signaling. Parreira et al. found that the *PPP2R2D* variant (rs34473884), which was significantly correlated with the DISH/CC phenotype, is the likely cause of the condition’s development [15].

#### 2.1.6. BMP4

BMP4 is a member of the BMP family and the TGF-β superfamily. It is involved in osteoblast differentiation and bone formation, and is a factor that stimulates soft bone ossification and the healing of bone fractures at an early stage. BMP4 was also found in mesenchymal cells, soft bone cells, the periosteum, bone-marrow cavities, and muscle cells adjacent to a fractured bone. BMP4 can indirectly stimulate bone formation by inhibiting osteoclastogenesis.

The increased expression of *BMP2* and *BMP4* mRNA was observed as a result of mechanical stress in spinal ligament cells in OPLL patients, and it is therefore possible that this pathway may also be involved in ossification in DISH [5]. A significant association was identified between DISH/CC and the genetic variant in *BMP4* (rs17563) [7].

#### 2.1.7. ALK2

Activin type I receptor (ALK2) is a transmembrane serine kinase receptor that activates intracellular signaling pathways when bound to BMP. The p.K400E mutation in the *ALK2* gene was demonstrated in a patient with DISH. Overexpressing cells ALK2 p.K400E activate BMP signaling in response to osteogenic BMP ligands and a lack of response to non-osteogenic ligand - activin A. BMP signaling ALK2 p.K400E was further enhanced by the coexpression of the BMP type II receptor (BMPR-II) that increased phosphorylation of ALK2 p. K400E. Increased ossification in DISH may be due to the enhanced induction of osteogenic BMP signaling (more than activin A) by the ALK2 p.K400E receptors enhanced by BMPR-II [21,22].

#### 2.1.8. ENT1

Type 1 equilibrative nucleoside transporter (ENT1) is responsible for most of the transport of adenosine across the cell membrane, which plays a role in bone homeostasis [23]. Adenosine promotes bone formation and resorption by activating different molecular pathways depending on which adenosine receptor is activated [24].

Mice lacking ENT1 (ENT1-KO) exhibited decreased adenosine uptake, increased plasma levels of circulating adenosine, and progressive ectopic calcified tissue of the spine and the annulus fibrosus of the intervertebral disc [20]. Studies were carried out on age-matched wild-type (WT) and ENT1-KO mice, and human cadaver spines meeting radiographic DISH criteria. The role of ENT1 in bone metabolism in vivo may be confirmed by the fact that patients without ENT1 exhibit increased ectopic mineralization [25].

Micro-computed tomography (CT) ENT1-KO mice revealed ectopic tissue calcification in the spine cervical–thoracic section. Histological examination of human DISH changes in mice resembled ENT1-KO. Microarray analysis in the affected tissue of ENT1-KO mice revealed extensive transcription dysregulation of genes related to the cell cycle and genes involved in the regulation of bone mineralization and development [26].

This phenomenon may explain how high levels of adenosine reduce the sensitivity of adenosine receptors involved in the regulation of osteoclast and osteoblast functions [27].

#### 2.1.9. Epigenetics in DISH

Epigenetic changes in the Wnt pathway genes are associated with changes in bone density and osteoblast function [28]. DNA methylation is an important gene regulator of the Wnt pathway. The Wnt ROR2 coreceptor shows progressive reduction in the methylation of its promoter DNA as it matures from mesenchymal stem cells into differentiated osteoblasts. The hypomethylation of ROR2 and WNT5a was demonstrated in the mesenchymal stem cells of isolated spinal ligaments in patients with DISH. This hypomethylation was associated with increased expression of ROR2 and WNT5a [29].

Noncoding RNA (ncRNA) includes microRNA (miRNA), long noncoding RNA (lncRNA), and circular RNA (circRNA), which regulate neoplastic, inflammatory, and degenerative processes [30]. miRNAs are short noncoding RNAs that are about 22 nucleotides in length and bind to complementary targets in the three untranslated regions (UTRs) of messenger RNA (mRNA), inhibiting mRNA translation and regulating many signaling pathways [31]. The level of miRNA expression varies by tissue and fine-tunes gene expression [30]. miRNAs interact with circRNA and lncRNA, regulating their stability; in turn, they bind to miRNAs, alleviating the inhibition of miRNAs on their target genes and increasing the expression levels of target genes [31]. The participation of noncoding RNA in ossification of spinal ligament was demonstrated. miR-563 interacts with SMURF1, which contributes to the ossification of OPLL by impeding ubiquitin-independent Smad degradation [32]. The role of miR-615-3p and miR-132-3p is to inhibit osteogenic differentiation by targeting forkhead box O1 protein (FOXO1) and growth differentiation factor 5 (GDF5) [32]. Angio miR-494 and miR-126-5p contribute to the proangiogenic effects of BMP4 on endothelial cells [33]. However, ncRNA coregulation between OPLL and DISH was not described.

### 2.2. Metabolic Factors

Growth hormone and insulin-like growth factor 1 were shown to promote bone formation and found to be elevated in patients with DISH [5]. The proliferation of cells forming fibroblasts, myoblasts, and osteoblasts is stimulated by TGF-β1, insulin, and bone morphogenic protein (BMP2). In DISH, the mechanisms of new bone growth, especially in tendon-attachment areas, are not known, and the possible signaling pathways involved in this process are Wnt-β catenins, nuclear factor κB, BMP2, prostaglandin I2, and endothelin1 [34].

Obesity correlated with DISH may be a factor in the pathogenesis of spinal calcification through a chronic process and inflammatory mediators (IL-6, TNF alpha, TGF-beta, VEGF) due to the hypertrophy and ossification of spinal ligaments. Obesity may also significantly affect the peripheral activity of osteoblasts and osteocytes through centrally regulated processes. Leptin (adipokine) acts as a cytokine and hormone in bone metabolism and cartilage homeostasis. As a proinflammatory cytokine, it stimulates the reorganization of the chondrocyte cytoskeleton by increasing the secretion of degradation mediators, and takes part in intervertebral-disc (IVD) tissue homeostasis by stimulating the proliferation of intervertebral disc cells and inhibiting the apoptosis of nucleus cells. In degenerative intervertebral disc disease (DDD), an increase in leptin was shown in IVD and the ligamentum flavum. Leptin mediating the inflammatory response of IL-6 causes hypertrophy and fibrosis of the yellow ligament by increasing collagen production. Leptin is expressed in the yellow ligament and adjacent fat-favoring epidural fibrosis yellow ligament. Adiponectin is an adipokine with an anti-inflammatory effect that reduces the production of TNF alpha, and it is less concentrated in DDD. In addition, the control of fat in bone regulation showed caloric state-dependent responses in central neuropeptide Y (NPY). In states where high-calorie NPY decreases rapidly, it increases the activity of osteoblasts [35].

Sohn et al. demonstrated an increase in bone mineral density (BMD) of the lumbar spine and the femoral neck in patients with DISH compared to the control group. The number of spine levels covered by DISH correlated with the BMD of the lumbar spine. These results may suggest that, in DISH, the increase in BMD is a consequence of metabolic processes [36].

#### 2.2.1. Metabolic Syndrome and Obesity

Mader et al. found statistically significantly increased waist circumference and BMI in patients with DISH compared to those of the control group, regardless of gender, which indicates an association with obesity [37]. There were no significant differences between groups in serum total cholesterol, high-density lipoprotein, low-density lipoprotein, or serum triglycerides [37,38].

#### 2.2.2. Diabetes

There is increased incidence of DISH in people with Type 2 diabetes and impaired glucose tolerance [39,40]. The Wnt signal pathway is regulated by inhibitors sclerostin and Dickkopf-related protein-1 (Dkk-1). Patients with DISH showed lower levels of DKK-1 than those of healthy people [39].

#### 2.2.3. Gout

Although DISH is more often diagnosed in patients with gout, genotypic analysis related to gout did not confirm that it was a cause of DISH. Most likely, gout and DISH share common risk factors, such as metabolic syndrome and obesity [41].

### 2.3. Vascular Factors

A significant increase in the number and width of the vertebral body nutritional orifices and hypervascularization was observed in people with DISH [42]. However, it is not known whether the newly formed bone required more supplying blood vessels or the growth of the blood vessels facilitated or caused new bone to form.

The process of heterotopic new bone formation in the tendon attachment areas of DISH patients is associated with abnormal osteoblast growth and activity, with active bone remodeling. Osteogenesis is initiated by the growth factors insulin, insulin-like growth factor-1, and growth hormone. Their action is not limited to bone tissue alone, and it is not clear why ossification only begins in certain places. One possible explanation for this selective localization is angiogenesis, which is essential for osteoblast proliferation. This may be due to the complex pathway of intercellular signaling between the vascular endothelium and bone cells involving numerous mediators such as VEGF, the primary fibroblast growth factor, TGF-β, and PDGF. This vascular endothelial mediation mechanism enables the targeting of osteoclast and osteoblast precursors to specific locations. These processes are also regulated by mediators and bone regulators including cytokines, estrogen, and parathyroid hormone (PTH). Angiogenesis is an important factor in metabolic syndrome, visceral obesity, dyslipidemia, diabetes, and atherosclerosis; the association of metabolic disorders with DISH may support the role of angiogenesis. In patients with DISH, a higher incidence of aortic-valve sclerosis (a marker of atherosclerosis and cardiovascular events) and bonelike arterial calcification was found. Due to the angiogenesis stimulating activity in patients with DISH, the disease can be considered part of the syndrome, not the disease. Metabolic disorders accompanied by DISH, and their common characteristic is angiogenesis [43].

Another vascular aspect was investigated by Bakker et al., who showed that newly formed bone in the DISH of the cervical spine is located symmetrically in front of the vertebral body as opposed to the thoracic spine, where it is located asymmetrically on the anterolateral surface of the vertebra. Arteries can act as a natural barrier to newly formed bone in DISH, so major vessels in the neck lateral to the vertebral bodies and the absence of segmental arteries in the cervical vertebrae can contribute to linear (rodlike) bone growth, as opposed to the thoracic spine, where segmental vessels are present. Unlimited forward bone formation may explain the ventral displacement of the trachea and esophagus [44].

### 2.4. Inflammatory Factors

Some authors suggest the inflammatory factors characterize DISH, but so far, no scientific evidence was found [6]. Histological examinations of human DISH patients compared to ENT1-KO mice showed no inflammatory character [26].

## 3. Clinical Aspects

### 3.1. Symptoms

Most cases of cervical-spine DISH are asymptomatic or only slightly symptomatic. Symptoms related to the locomotive system include neck pain and stiffness. Compression of the throat, esophagus, and trachea can cause dysphagia, shortness of breath, and stridor (Table 2) [45]. Symptomatic cases are more common in men, and the main complaint is dysphagia (75%). A case of the conversion of asymptomatic DISH to symptomatic DISH as a result of an upper-respiratory-tract infection was described [46]. This may be relevant in view of the appearance of respiratory viruses such as coronavirus disease 2019 (COVID-19), which causes severe mucositis [46]. The management of DISH with airway disorders should focus on intubation or tracheostomy in the event of an emergency that requires intervention [47].

### 3.2. Diagnostics

X-rays of the cervical spine are usually sufficient for diagnosis, but computed tomography (CT) and magnetic resonance imaging (MRI) help to determine the extent of the hyperostosis and its location in relation to the esophagus, and to identify associated changes and complications (e.g., spinal stenosis and compression myelomalacia) [53,54]. Radiologically diagnosed DISH is based on the presence of “fluid” ossification along the anterolateral margins of at least four adjacent vertebrae, and no lesions associated with spondyloarthropathy or degenerative spondylosis.

Hyperostosis occurs along the lower half of the anterior edge of the vertebral body in the form of a “drooping drop”. The “drops” then tend to grow to form spurs (enthesophytes) until they band together. Spurs widen on the upper and anterior segment of the vertebra, resulting in a “candle flame”, “parrot’s beak”, or “bridge” [55].

Esophageal diagnosis and laryngoscopy combined with a fluoroscopic barium swallowing test may be useful in the diagnosis of DISH contributing to dysphagia or dysphonia [56].

Videofluoroscopy (videoesophagogram) is considered to be the gold standard to show the exact relationship of changes in the cervical spine with the swallowing function in patients with dysphagia [50,57].

Criteria for diagnosis in research and clinical settings are controversial. In the research setting, it is recommended to completely exclude DISH cases from patients with comorbidities described in the Resnick and Niwayam exclusion criteria [4,58].

### 3.3. Treatment

Initially, all mild-to-moderate cases are treated with conservative methods, such as diet modification by changing eating habits to a softer diet, drinking plenty of water with food, eating more frequent and smaller meals, and taking more time to complete a meal [41]. Painkillers, sedatives, nonsteroidal anti-inflammatory drugs, muscle relaxants, and anti-reflux medications are also used [5]. The role of disodium etidronate bisphosphonate in preventing DISH in animal testing is not yet a standard in humans [56]. Corticosteroid injections are also an option [5].

Physiotherapy, aimed at maintaining mobility and relieving pain, also has its place as a therapy. As an accompaniment to degenerative changes, instruction on joint-relief techniques may be important [5].

Due to the association of DISH with metabolic changes and visceral obesity, the education and implementation of a healthy lifestyle in the form of physical activity, low consumption of saturated fat and carbohydrates, and weight reduction are important [59,60].

The indication for surgical treatment is worsening airway obstruction and/or dysphagia with failed ambulatory therapy [61]. Surgical intervention is reserved for symptomatic cases that do not respond to nonsurgical treatment, progressive dysphagia with unintentional weight loss, and people with respiratory symptoms [45].

**Figure 2:**
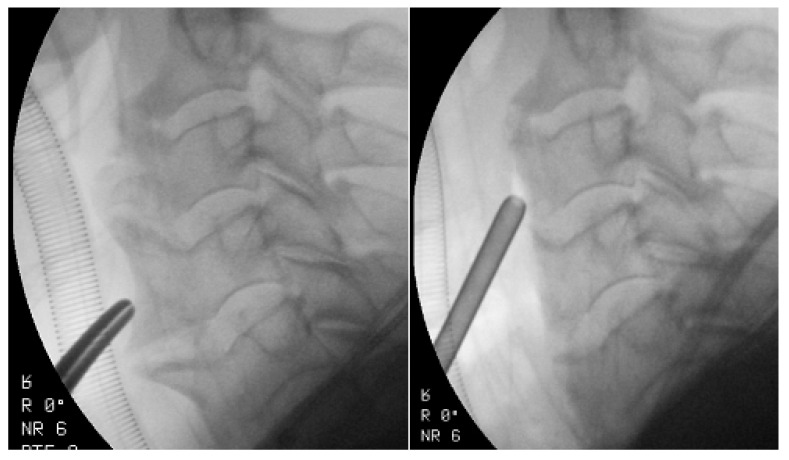
Intraoperative fluoroscopic imaging before and after osteophyte removal in a 72-year-old patient with DISH with dysphagia.

Surgical treatment involves the direct anterior removal of osteophytes (Figure 2) [62]. Surgical resection of osteophytes usually results in excellent symptom relief [53]. Spinal fusion may sometimes be warranted if there are accompanying symptoms of cervical-spine instability [63]. Spinal fusion should be considered in patients with concurrent spinal-cord or nerve compression, or spinal instability. Approximately 10% of patients with DISH associated dysphagia require surgical decompression, while 70–100% of patients report improvement in dysphagia after the surgical resection of anterior cervical-spine osteophytes [64]. Worse results were reported in older patients (>75 years) [61,64]. Large osteophytes increase the risk of damage to the esophagus during surgical exposure. The esophagus can be difficult to mobilize, and to some extent, adhere to other cervical fascia due to a local inflammatory response. The complication of the procedure concerns the esophagus and recurrent damage to the laryngeal nerve [65]. Reossification with the formation of new osteophytes may occur, and a second operation may be indicated [50]. DISH recurrence of the cervical spine may slowly and progressively develop, with an average rate of 1–2 mm/year [61,66].


The pathogenesis of DISH is unclear: polygenetic and epigenetic factors associated with metabolic disorders, with the probable contribution of genes from signaling pathways involved in bone formation (WNT, TGF β). DISH is usually asymptomatic.DISHphagia: the failure of conservative treatments (pharmacotherapy, modification by changing eating habits, physiotherapy) is an indication for the surgical removal of the osteophyte.


## 4. Summary

DISH is a common asymptomatic disease characterized by new bone formation and metabolic abnormalities. We still know little about the pathogenesis of DISH; therefore, there is no possibility of causal treatment. Clinical and metabolic disorders in DISH are associated with extensive proliferative processes in the musculoskeletal system. DISH pathogenesis is polygenic, and influenced by the interaction of many gene variants and environmental factors. The DISH phenotype consists of potential disturbances in various genes with different chromosomal localizations and expressions. Further molecular studies are required to clarify and verify the diagnostic criteria, with particular emphasis on the early symptoms. Studying the molecular mechanisms involved in bone formation, along with identifying the genetic markers associated with DISH, may help to discover the ossification pathogenesis of ligaments and tendon attachments; this could lead to targeted and effective therapies.

## Figures and Tables

**Table 1:** Genetic variants associated with DISH.

Gene	SNP	Population	Disorder Associated with DISH	References
*FGF2*	rs1476217/rs3747676	Korean	OPLL	Jun et al. [15]
*COL6A1*	intron 32 (−29)	Japanese	OPLL	Tsukahara et al. [16]
*PPP2R2D*	rs34473884	Azorean	CC	Parreira et al. [14]
RSPO4	rs146447064, rs14915407	Azorean	CC	Couto er al. [13]
BMP4	rs17563	Azorean	CC	Couto er al. [13]
LEMD3	rs201930700	Azorean	CC	Couto er al. [13]

SNP, single nucleotide polymorphism; OPLL, ossification of the posterior longitudinal ligament; CC, chondrocalcinosis.

**Table 2:** Symptoms of cervical-spine DISH.

Symptom	Characteristics and Frequency	Mechanism
Neck pain, radicular pain, and stiffness.	Frequently, motor segments are C5/C6 (40%) followed by C4/C5.	Increasing degeneration of intervertebral discs and ossification of posterior longitudinal ligament may lead to narrowing of spinal canal.
Dysphagia (DISHphagia)	Present with solid foods, improved by neck flexion, and aggravated by neck straightening [48]. Incidence in patients with DISH is in the range of 0.2–28% [49]. Combined symptoms: foreign-body sensation, odynophagia, saliva stagnation, dysphonia, dyspnea [50]. Incidence of dysphagia in patients with cervical-spine DISH was influenced by osteophyte thickness, cervical-spine mobility, and craniocervical position [48].	Lower cervical-spine osteophytes on levels C4–C6 can cause esophageal stricture and mechanical trouble, causing varying degrees of esophageal obstruction, impaired motility of the epiglottis, and larynx-cartilage deformity [51]. Large osteophytes can lead to dysphagia through direct mechanical obstruction; smaller osteophytes can act at the sites accompanying immobilization of the esophagus because the esophagus is anatomically anchored on the cricoid-cartilage and diaphragm level. Chronic pressure may cause inflammatory reaction in the wall of the esophagus and adjacent soft tissue, leading to fibrosis and adhesions, and painful reflex esophageal-spasm-induced irritant osteophyte [52]. Recurrent nerve paralysis caused by hyperostosis may aggravate narrowing [50].
Breathing disturbances	Concomitant symptoms: dyspnea (14%), cough (3%), dysphonia (2.5%), and stridor [3,47].	Upper cervical-spine osteophytes are responsible for the oropharynx, causing respiratory distress and stridor. Respiratory symptoms related to DISH are associated with osteophytes inflammation and fibrosis of the esophageal wall and adjacent tissues causing obstruction of the upper respiratory tract, and direct lesion of the recurrent laryngeal nerve [3,47]

## Data Availability

MDPI research data policies.
